# On a New Mode of Reducing Prolapsus Uteri

**Published:** 1854-08

**Authors:** Reuben Searcy

**Affiliations:** Tuscaloosa, Alabama


### ART. II.—ON A NEW MODE OF REDUCING PROLAPSUS UTERI.

BY REUBEN SEARCY, M. D., OF TUSCALOOSA, ALABAMA.

MR. EDITOR:—I am not aware that any writer has recommended precisely my manner of operation for the correction of the misplacement of the organ now under consideration. As I have been uniformly successful, I thought, probably, that by this communication, I might make some suggestions that may lead physicians to a more easy and successful operation than is usually practiced.—First make a digital examination, to ascertain the condition of the womb; if you find it engorged with a dipping down into the vagina; place the female on her knees, her breast low on the bed, she should bend her back as far forward as possible; which I term sway the back; by this position the weight of the bowels, and the abdominal muscular contractions are taken off, and the operator has every chance of replacing the organ; by introducing the index and middle fingers, and reaching, by pressing his fingers as far into the vagina as possible, the body of the womb lying next to the rectum; then by a firm pressure, carry the womb to its proper place. If the womb has been long out of place, it will take much more force than is generally imagined to effect a restoration; which must be accomplished, let the pain be ever so great; when this is accomplished the female should be directed to fall on her side without rising up, and remain in a horizontal position several hours, or until next morning, as I generally select the usual bed time for the adjustment. I allow my patient to be up and take exercise to invigorate the system, giving directions to avoid lifting, and severe exertion, or any thing that would cause a straining of the abdominal muscles, and for her to lie down frequently and every time on lying down, to place herself on her knees, breast down and back swayed, and while in this position to make a suction or upward drawing of the abdominal muscles; this will bring the womb that would otherwise be disposed to fall as low as first found, back into its proper place, this position and practice should be especially insisted on every night on retiring.

Frequently one replacement, by following up the practice of position, together with the suction and swaying of the back, and for her, after a few suctions to remain on her knees, in a perfectly relaxed condition of all the muscles, before tumbling down for the night. By this practice the womb will soon accomodate itself in its proper place, and the ligaments and other parts sustaining the womb, will strengthen.

If the symptoms are such after a few days, as to cause the practitioner to suspect that the womb is not right, examine again as before, etc. Generally it is not necessary until after the next catamenia has ceased, then it would be well to re-examine, etc. If, on the first examination, the womb is found retroverted, after placing the woman on her knees, breast down and back swayed; then the doctor will proceed; and unless he has unusually long fingers he will not be able to reach the fundus of the womb by the vagina; then it will be necessary for him to explain to her the necessity for him to introduce the finger, (the index) into the rectum; then by running the finger high up in the bowel, reach to where the fundus of the womb is pressing against the bowel; then by a very firm pressure forward, the bowel going before the finger, the womb is carried in a proper line for adjustment, as before directed; this breaking up or breaking out of its unnatural bed or displacement is extremely painful, and a timid practitioner will desist through sympathy for the sufferer before he accomplishes his object, and leave his patient to drag out a life of suffering, unless she should fortunately fall into bolder hands. The suffering for a few moments should not deter the operator from pushing to the accomplishment of so great and so permanent relief.

It will be readily perceived that I discard all pessaries. I deem them irritants and excitants, and seldom do good, and therefore should be condemned and discarded from this practice.

By my plan, the patient exercises sufficiently for her general health, she is not subjected to so much manipulation, as she is on the other plans. The plan presents itself at once forcibly to the minds of all, as the most natural and easy of accomplishment over all others.

Abdominal or bowel supporters might be necessary for some females to wear, where the bowels press too heavily on the broad ligament or floor of the abdomen in which the womb is suspended. The patient should be directed not to let the bladder become much distended.

All departures from general health should have, medicinally, their appropriate attention.

